# Baicalein Rescues Delayed Cooling via Preservation of Akt Activation and Akt-Mediated Phospholamban Phosphorylation

**DOI:** 10.3390/ijms19040973

**Published:** 2018-03-24

**Authors:** Zuohui Shao, Sy-Jou Chen, Xiangdong Zhu, Chunpei Lee, Hsien-Hao Huang, Angelo Meliton, Changqing Li, Terry L. Vanden Hoek, Jing Li

**Affiliations:** 1 Center of Advanced Resuscitation Medicine, Center for Cardiovascular Research, Department of Emergency Medicine, University of Illinois Hospital, and Health Sciences System, Chicago, IL 60612, USA; zshao42@gmail.com (Z.S.); syjou.chen@mail.ndmctsgh.edu.tw (S.-J.C.); xiangdon@uic.edu (X.Z.); pheanys@uic.edu (C.L.); hhhuang@vghtpe.gov.tw (H.-H.H.); cqli41s@gmail.com (C.L.);; 2 Department of Emergency Medicine, Tri-Service General Hospital, National Defense Medical Center, Taipei 114, Taiwan; 3 Department of Emergency Medicine, Taipei Veterans General Hospital and Emergency Medicine, College of Medicine, National Yang-Ming University, Taipei 112, Taiwan; 4 Department of Medicine, Section of Pulmonary and Critical Care Medicine, University of Chicago, Chicago, IL 60637, USA; ameliton@uchicago.edu

**Keywords:** intraischemic cooling, delayed cooling, baicalein, ischemia/reperfusion, reactive oxygen species (ROS), cardiomyocyte, Akt, cell death, contractility

## Abstract

Cooling reduces the ischemia/reperfusion (I/R) injury seen in sudden cardiac arrest (SCA) by decreasing the burst of reactive oxygen species (ROS). Its cardioprotection is diminished when delay in reaching the target temperature occurs. Baicalein, a flavonoid derived from the root of *Scutellaria baicalensis Georgi*, possesses antioxidant properties. Therefore, we hypothesized that baicalein can rescue cooling cardioprotection when cooling is delayed. Two murine cardiomyocyte models, an I/R model (90 min ischemia/3 h reperfusion) and stunning model (30 min ischemia/90 min reperfusion), were used to assess cell survival and contractility, respectively. Cooling (32 °C) was initiated either during ischemia or during reperfusion. Cell viability and ROS generation were measured. Cell contractility was evaluated by real-time phase-contrast imaging. Our results showed that cooling reduced cell death and ROS generation, and this effect was diminished when cooling was delayed. Baicalein (25 µM), given either at the start of reperfusion or start of cooling, resulted in a comparable reduction of cell death and ROS production. Baicalein improved phospholamban phosphorylation, contractility recovery, and cell survival. These effects were Akt-dependent. In addition, no synergistic effect was observed with the combined treatments of cooling and baicalein. Our data suggest that baicalein may serve as a novel adjunct therapeutic strategy for SCA resuscitation.

## 1. Introduction

Ischemia/reperfusion (I/R) injury is often seen in sudden cardiac arrest, resulting in early myocardial contractility impairment and later cell death if the ischemia is prolonged. Both of these events are the results of stunned myocardium that consists of a burst of reactive oxygen species (ROS), impaired metabolism, imbalance of calcium homeostasis, and alteration of contractile protein structures [1,2,3,4,5,6]. Cooling, one of the few treatment strategies, has been proven to be a cardioprotective approach in improving neurologically intact survival after sudden cardiac arrest (SCA), which affects 350,000 people each year in the USA, with an average survival rate of about 7% [7]. One of the factors influencing cooling protection is optimal timing of cooling [8,9]. Our prior work demonstrated that intraischemic cooling (IC) was most protective against I/R injury [10,11]. Similar benefit was also observed in SCA clinically. However, due to the difficulties in reaching the target temperature, even in lowering temperature by a few degrees, cooling is often delayed, which can diminish its cardiac protection [12]. Therefore, an agent mimicking the cooling effect which can be used in the context of delayed cooling would be highly beneficial and translational.

Baicalein (5,6,7-trihydroxyflavone) is a flavonoid and one of the main compounds derived from the dried roots of *Scutellaria baicalensis Georgi* (Huangqin). It possesses a multitude of bioactivities, such as antioxidant, antiapoptosis, and antiinflammation. Its protective role against chronic diseases such as cancers and acute ischemia/reperfusion (I/R) injuries have been evidenced. It has been widely used as a dietary supplement with great therapeutical actions. The antioxidant properties of baicalein have been well studied in both in vitro and in vivo. It scavenges ROS, including hydroxyl radicals (^•^OH), superoxide (O_2_^•−^), and hydrogen peroxide (H_2_O_2_), in addition to its property of acting as an iron chelator that inhibits the conversion of hydrogen peroxide to hydroxyl radicals during I/R injury, and reduces the oxidation of nucleic acids and proteins [13,14]. Our previous work showed that baicalein improved cardiomyocyte survival via its oxidant scavenging property [15,16] and its ability to activate Akt [17], similar to cooling protection against I/R injury in in vitro and in vivo studies [18,19,20,21]. However, it remains unknown whether baicalein can rescue the cardioprotection of cooling when it is delayed. Thus, in this study, we extended our previous finding to investigate whether baicalein improves cell contractility and survival in the setting of delayed cooling. We also aimed to study the optimal timing of baicalein administration and the mechanisms of baicalein rescue benefits in the context of delayed cooling.

## 2. Results

**Figure 1:**
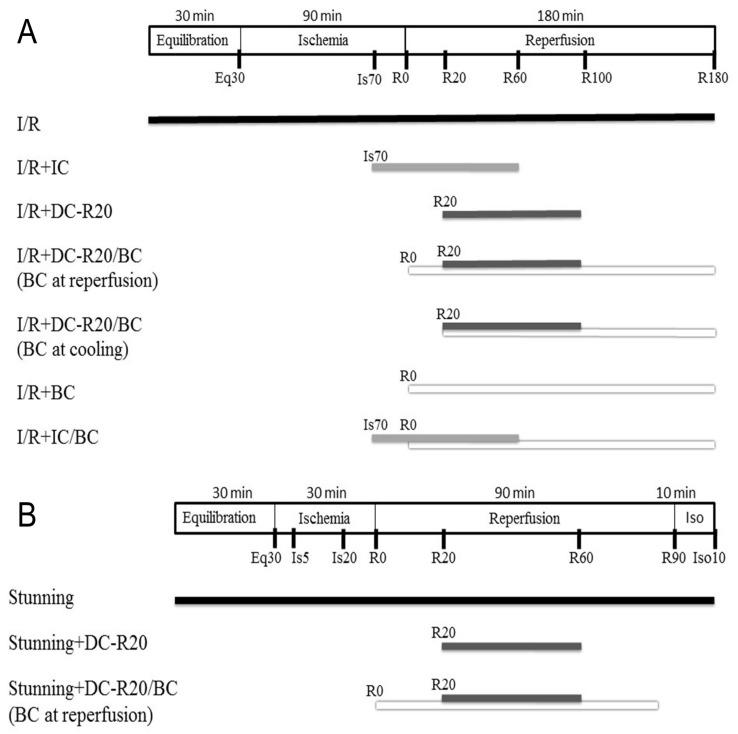
Schematic diagram of ischemia/reperfusion (I/R) and stunning protocols. (**A**) I/R model: mouse cardiomyocytes were equilibrated (Eq) for 30 min and then subjected to 90 min of simulated ischemia (Is) followed by 180 min of reperfusion (R). Intraischemic cooling (IC, 32 °C) was initiated at the last 20 min of ischemia and continued into 60 min of reperfusion for a total of 80 min. Cells then rewarmed to 37 °C. Delayed cooling (DC) was initiated at 20 min of reperfusion. (**B**) Stunning model: cells were equilibrated for 30 min, and then subjected to 30 min of simulated ischemia and 90 min of reperfusion followed by 10 min of isoproterenol. IC was initiated at the last 10 min of ischemia and ended 60 min of reperfusion, and then cells were rewarmed to 37 °C for 30 min. Baicalein (BC, 25 µM) was administrated at the start of reperfusion.

Two mouse cardiomyocyte models of I/R (longer ischemic time, 90 min) [11] and stunning (shorter ischemic time, 30 min) [22] were used in this study. The I/R model was used to evaluate the cell viability, while the stunning model was used to assess the contractility alteration (no significant cell death occurs). Details of the protocols and experimental groups are described in Materials and Methods 4.3 (Figure 1). In brief, the experiment groups included: (1) I/R; (2) I/R + intraischemic cooling (I/R + IC); (3) I/R + delayed cooling R5 (DC-R5); (4) I/R + delayed cooling R10 (DC-R10); (5) I/R + delayed cooling R15 (DC-R15); (6) I/R + delayed cooling R20 (I/R + DC-R20); (7) I/R + DC-R20/baicalein (BC); (8) DC-R20/BC-C; (9) I/R + BC alone; (10) I/R + IC/BC; (11) DC-R20/BC + API-2 (an Akt inhibitor).

### 2.1. Delayed Cooling-Diminished Effects of Intraischemic Cooling on Cell Death and ROS Generation

**Figure 2:**
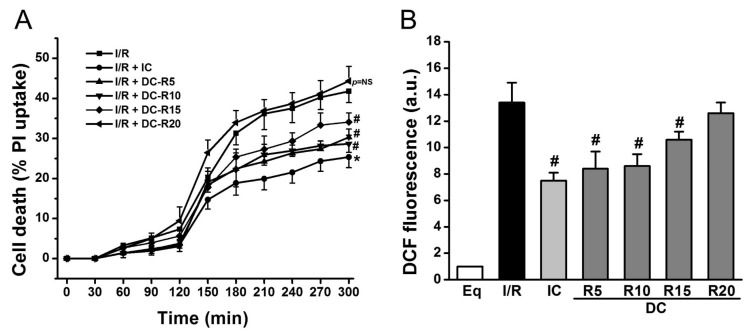
Cooling protective effects were lost when its delay was prolonged. I/R-induced cell death was attenuated by intraischemic cooling (IC). (**A**) Cooling, initiated at reperfusion of 5 min (DC-R5), 10 min (DC-R10), and 15 min (DC-R15), decreased cell death. The delayed cooling, initiated at reperfusion of 20 min (DC-R20, referred to as DC), was unable to reduce cell death. Cell death at R180 was assessed by propidium iodide (PI, 5 μM). (**B**) I/R-induced ROS generation measured by DCF fluorescence was attenuated by IC. Cooling benefit was lost when it was initiated at R20. * *p* < 0.01 and ^#^ *p* < 0.05, *n* = 6 for panel A, and *n* = 5 for panel B. Data are expressed as mean ± SE.

To determine the window of intraischemic cooling protection, we examined the following treatment groups: (1) I/R; (2) I/R + IC; (3) I/R + DC-R5; (4) I/R + DC-R10; (5) I/R + DC-R15; (6) I/R + DC-R20. I/R alone resulted in a significant cell death at reperfusion 180 min (R180), which was reduced by IC (32 °C) (41.8 ± 2.8% vs. 25.4 ± 2.6% in IC, *p* < 0.01). Cell death was decreased even when cooling was delayed, and started at 5 (DC-R5, 27.3 ± 2.0% vs. 41.8 ± 2.8%, *p* < 0.01), 10 (DC-R10, 28.7 ± 0.9% vs. 41.8 ± 2.8%, *p* < 0.01), and 15 min (DC-R15, 34.1 ± 2.3% vs. 41.8 ± 2.8%, *p* < 0.05) of reperfusion, as shown in Figure 2A, respectively. However, when cooling was delayed further and started at 20 min of reperfusion, it failed to reduce cell death (44.3 ± 3.7% vs. 41.8 ± 2.8%, *p* = NS, not significant) (Figure 2A). Next, ROS generation was measured by carboxy-dichlorofluorescein (DCF) at R90 in these protocols and presented in Figure 2B. I/R triggered a striking increase in ROS production (13.4 ± 1.5 arbitrary units (a.u.) vs. 1.0 ± 0% in Eq, *p* < 0.01). Compared to I/R alone, ROS generation was markedly decreased by IC (13.4 ± 1.5 a.u. vs. 7.5 ± 0.6 a.u. in IC, *p* < 0.01). Cooling did not lower ROS production as much as IC when it was initiated later (8.4 ± 1.3 a.u. in DC-R5; 8.6 ± 0.9 a.u. in DC-R10; 10.6 ± 0.6 a.u. in DC-R15, *p* < 0.05). The cooling effect on reducing ROS generation was diminished when it started at 20 min of reperfusion (12.6 ± 0.8 a.u. in DC-R20 vs. 13.4 ± 1.5 a.u. in I/R alone, *p* = NS). These results suggest that the timing of cooling is a critical determinant of its cardioprotection: the earlier that cooling is initiated, the less cardiomyocyte death occurs. Furthermore, cooling protection is related to its ability to reduce ROS generation. Based on these results, the DC-R20 protocol was chosen in subsequent studies and DC-R20 was referred to as DC.

### 2.2. Baicalein Rescued the Delayed Cooling When Administered at Both the Start of Reperfusion and the Initiation of Cooling during Reperfusion

**Figure 3:**
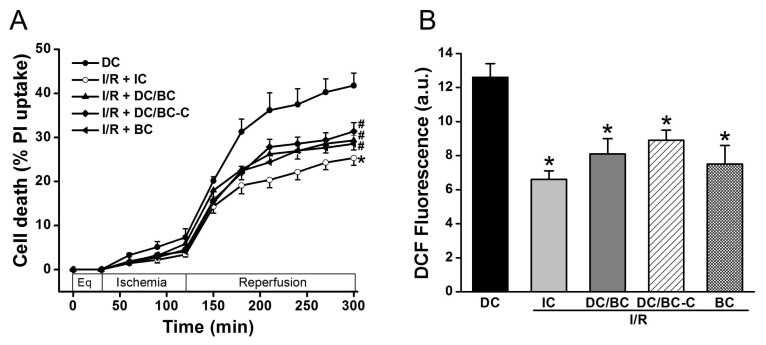
Baicalein (25 µM) administration at either the start of reperfusion (DC/BC) or the initiation of cooling (DC/BC-C) reduced cell death (**A**) and ROS generation (**B**). Baicalein alone resulted in comparable effects to both DC/BC and DC/BC-C on cell death and ROS. * *p* < 0.01, ^#^ *p* < 0.05, *n* = 6. The data are expressed as mean ± SE.

To test the effectiveness of baicalein (BC) and the timing of baicalein administration on cell death and ROS generation in the context of delayed cooling, baicalein (25 µM) was given either at the start of reperfusion (DC/BC) or at the initiation of cooling (DC/BC-C). As depicted in Figure 3A, compared to DC, IC again reduced cell death (25.3 ± 1.6% vs. 41.7 ± 2.8% in DC, *p* < 0.01). Both DC/BC and DC/BC-C decreased cell death with no noticeable difference between these two treatments (29.3 ± 2.2% or 31.4 ± 2.0% vs. 41.7 ± 2.8% in DC, *p* < 0.05). Baicalein alone given at the start of reperfusion resulted in a decrease in cell death from 41.7 ± 2.8% to 28.5 ± 1.9% (*p* < 0.05), and this effect was comparable to those of DC-BC or DC/BC-C. We next examined the effect of baicalein on ROS production. As presented in Figure 3B, a burst of ROS production measured by DCF fluorescence at R90 was observed with DC, and this increase was attenuated by IC, DC/BC, or DC/BC-C treatments (12.6 ± 0.8 a.u. vs. 6.6 ± 0.5 a.u. in IC; 8.1 ± 0.9 a.u. in DC/BC; and 8.3 ± 0.6 a.u. in DC/BC-C, *p* < 0.01). Similar to its effect on cell death, baicalein alone resulted in a reduction of ROS comparable to both DC/BC and DC/BC-C. These data suggest that baicalein protection against cell death is correlated with its ability to decease ROS generation, similar to cooling. With no significant difference between DC/BC and DC/BC-C on both cell death and ROS generation, DC/BC was used for subsequent experiments.

### 2.3. Baicalein Enhanced Contractility Recovery and Phospholamban Phosphorylation

**Figure 4:**
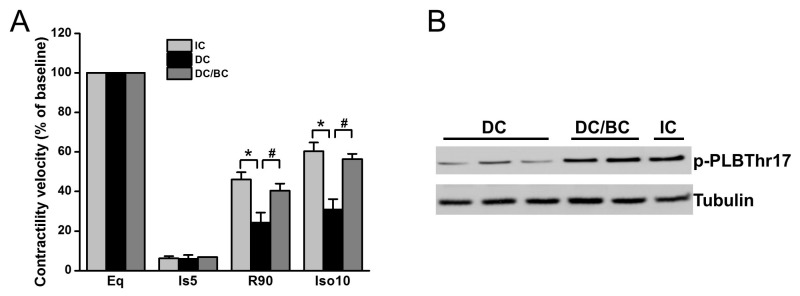
Baicalein improved cell contractile velocity and reserve capacity in the context of delayed cooling. Furthermore, it increased phospholamban phosphorylation. Cells were equilibrated for 30 min and then subjected to 30 min simulated ischemia, and 90 min reperfusion (R90) followed by 10 min 0f 10 µM isoproterenol (Ios10) to evaluate the reserve capacity of contractility activated by β-adrenergic stimulation. (**A**) Cell contractile velocity was measured at baseline (BL), ischemia 5 min (Is5), R90 and Iso10 by microscopy and analyzed by Matlab software. Baseline contractile velocity was presented as 100%. Baicalein increased both cell contractile velocity and contractile reserve, similar to IC (*n* = 6). (**B**) Baicalein and IC increased PLB phosphorylation (p-PLB) compared to DC (*n* = 4). * *p* < 0.01, ^#^ *p* < 0.05. Data are expressed as mean ± SE.

We previously demonstrated that cooling improved the recovery of synchronous cell contractile velocity following I/R injury, but recovery was lost when cooling was delayed [22]. Using the established cardiomyocyte stunning model, we tested whether baicalein can rescue cell contractile function in the condition of delayed cooling. Three groups were included: (1) IC; (2) DC; and (3) DC/BC. The contractile velocity was measured after each treatment and compared with the baseline, which was considered as 100%. As shown in Figure 4A, cell contraction was diminished within 5 min of ischemia. IC resulted in the recovery of cell contractile velocity at 90 min reperfusion to about 46% of the baseline, but delayed cooling only improved contractile velocity to 25%. Baicalein treatment in the context of delayed cooling enhanced myocyte contractile velocity, a similar improvement to that of IC. It also preserved the reserve capacity of contractility to 56% of the baseline upon supplementation of isoproterenol (10 μM) for 10 min, which is comparable to that of IC (about 60%). To further study the mechanisms of contractility recovery, cell lysates were collected after reperfusion of 30 min (R30) and analyzed by Western blot for phospholamban (PLB), a small protein regulating heart cell contraction. Increased phosphorylation of PLB indicates contractile function enhancement [23]. Compared to DC, PLB was significantly phosphorylated at PLBThr17, a site that is directly phosphorylated by Akt which is activated by baicalein or IC (Figure 4B).

### 2.4. Baicalein Protection in the Context of Delayed Cooling Was Mediated by Akt Phosphorylation

**Figure 5:**
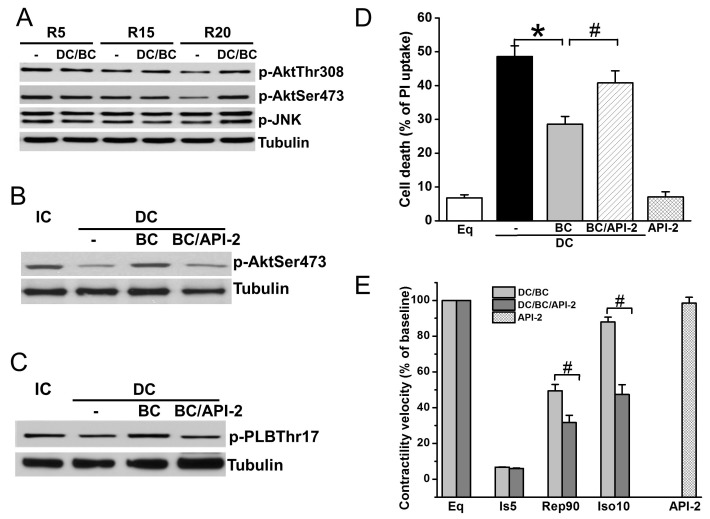
Baicalein rescues cooling benefit in the context of delayed cooling dependent on Akt activation. (**A**) DC resulted in a decrease of p-Akt (Thr308 and Ser473) at R30 as delay increases. It had no effect on p-JNK. (**B**) BC increased p-Akt which was blocked by pretreatment with API-2 (10 μM). (**C**) API-2 blocked BC-induced PLB phosphorylation. (**D**) API-2 blocked BC-induced cell survival. (**E**) API-2 blocked BC-induced contractility recovery and reserve. * *p* < 0.01, ^#^ *p* < 0.05, *n* = 3 for Western blot, *n* = 5 for contractile velocity. The data are expressed as mean ± SE.

Akt is a well-known prosurvival signaling, while JNK plays an important parallel role in the antisurvival signaling pathway [17,24]. To further understand the mechanisms of baicalein cardioprotection, cell lysates were collected after reperfusion of 30 min (R30) in DC protocols of R5, R15, and R20, and measured for phosphorylation of Akt (sites Thr308 and Ser473) and JNK (Thr183/Tyr185). As depicted in Figure 5A, Akt phosphorylation was diminished as the time of delayed cooling increased, which was preserved by baicalein treatment. In contrast, JNK phosphorylation was not affected by DC. Administration of Akt inhibitor API-2 one hour prior to ischemia abolished BC-induced Akt phosphorylation (Figure 5B) and phosphorylation of its downstream target PLB (Figure 5C). Accordingly, cell death (Figure 5D) and contractility recovery and reserve (Figure 5E) were blocked by API-2.

### 2.5. Combined Treatments of Baicalein and Intraischemic Cooling Exerts No Synergistic Effects

**Figure 6:**
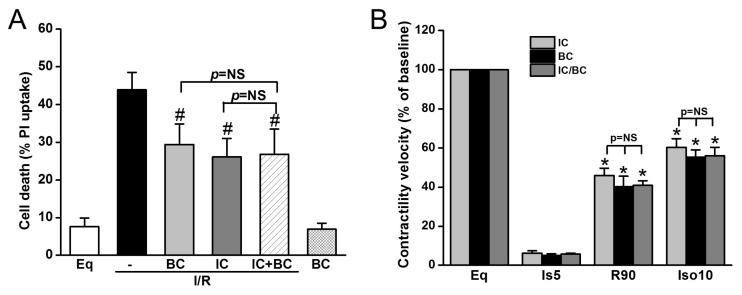
The combined treatments of intraischemic cooling (IC) and baicalein (BC) conferred no synergistic effects on cell survival and contractility recovery and reserve. Cells were treated with either IC or BC, alone or in combination. Both IC and BC alone similarly decreased I/R-induced cell death assessed at R180 (**A**) and cell contractile velocity and reserve measured at R90 (**B**). The combined treatments of IC and BC had no further protection on cell survival and cell contractility and reserve (*p* = NS). ^#^ *p* < 0.05, * *p* < 0.01; *n* = 4. The data are expressed as mean ± SE.

Additionally, we tested whether baicalein and IC treatment exerts synergistic effects on cell death and ROS generation in the context of delayed cooling. Six treatment groups were included: (1) Eq30; (2) I/R; (3) BC; (4) IC; (5) IC/BC; and (6) BC alone. Cells in groups 2–5 underwent I/R. while cells were not subjected to I/R in group 6. Compared to I/R alone (group 2), both BC and IC similarly decreased cell death. The combined treatments of BC and IC did not further reduce cell death, indicating the protection of BC and IC is mediated through similar mechanisms (Figure 6A). BC alone without I/R had no effect on cell survival. We further examined the effect of combined treatments of IC and BC on contractility recovery measured by contractile velocity. Similar to the results of cell survival, no synergistic effect was observed (Figure 6B).

## 3. Discussion

In this study, we demonstrated that intraischemic cooling benefit was diminished when the delay of cooling (32 °C) occurred, in two cardiomyocyte models: I/R and stunning. Baicalein administration at either the start of reperfusion or the initiation of cooling improved contractile recovery, in addition to its protection of cell survival in the context of delayed cooling. This protection is attributed to its ability to attenuate ROS generation, preserve Akt phosphorylation, and to increase Akt-mediated phospholamban phosphorylation.

Previous work has shown that I/R results in cell death. This is due, in part, to a burst of ROS generation during reperfusion, which triggers stress signaling cascades and leads to increased cell death [25,26]. Intraischemic cooling significantly attenuates ROS production, thereby decreasing cell death [11]. Clinically, cooling initiation occurs far later than resuscitation (such as cardiopulmonary resuscitation, CPR), and it often takes hours to reach the target temperature, even with mild cooling [27]. To better understand the effective treatment window of cooling, we simulated delayed cooling in mouse cardiomyocyte models of I/R and stunning by initiating cooling during reperfusion at 5, 10, 15, and 20 min. Although the cooling benefit was maintained when initiated before 15 min of reperfusion, its protection was lost when the delay was prolonged (Figure 2A), suggesting a narrow effective treatment window of cooling. This finding was in agreement with a rat stroke model in which a 20- to 30-min delay of cooling failed to provide neuroprotection due to irreversible cellular injury [28]. Loss of cooling protection was also noticed in cell contractility recovery, which markedly decreased when cooling was initiated at 20 min of reperfusion (Figure 4). Thus, cell rescue is time-sensitive. In clinical scenarios, intraischemic cooling has presented great benefits in improving the outcome of cardiac arrest. However, it is often delayed, which can negate the benefit of cooling [10,27]. The present study demonstrated that baicalein, a naturally occurring flavone, has the ability to rescue the protective effects of cooling. We have previously shown that pretreatment of baicalein attenuated ROS production [16], similar to intraischemic cooling [11]. In this study, when cooling was delayed and started at 20 min of reperfusion, significant ROS were generated and cell death was increased to levels similar to with no cooling (Figure 2B). Baicalein given at the start of reperfusion (DC/BC) or the start of cooling initiation (DC/BC-C) both inhibited ROS generation and cell death, with DC/BC being slightly better (Figure 3). Baicalein given at the start of reperfusion may not only prevent ROS generation, but also scavenge ROS that was already formed. Therefore, it resulted in slightly more reduction of ROS generation and cell death compared to baicalein given at the start of cooling initiation, which is caused at least in part by its scavenging property as reported previously by us [15]. This unique ability of baicalein that not only prevents free radical production, but also scavenges already-formed 1,1-diphenyl-2picrylhydrazyl (DPPH), superoxide, and hydroxyl radicals makes it an ideal candidate for treating ischemic injury as seen in cardiac arrest, especially in the situation of delayed cooling [14,15,16]. Whether this is a direct scavenging action of baicalein or an indirect effect via its inhibition of Fenton chemistry-induced radical damage was unclear, as baicalein can be a more potent antioxidant by chelating Fe^2+^ than by scavenging radicals [29]. Other mechanisms may also be involved in the downregulation of ROS by baicalein. Further, baicalein alone seemed to exert a similar reduction on cell death and ROS production, if not better than the combined treatment of baicalein in the context of delayed cooling. This raised the possibility that the administration of only baicalein may be sufficient to protect cardiomyocytes from I/R injury when cooling is delayed, and there may not be a need to even initiate cooling when it is not able to start early. However, this warrants further investigation, as cooling has demonstrated strong evidence for its efficacy of neuroprotection.

The current study also investigated the underlying signaling mechanisms of baicalein protection in the context of delayed cooling. Consistent with previous reports that baicalein treatment induces Akt phosphorylation [17,30], our study showed that Akt phosphorylation was decreased as the delay of cooling was prolonged, which was preserved with the treatment of baicalein given at the reperfusion. This may be related to its ability to decrease PTEN activity by preserving PTEN phosphorylation, leading to increased Akt phosphorylation and activation [28,30,31,32]. This may be in concert with baicalein’s ability to reduce ROS, leading to decreased interaction of oxidized Akt with PTEN, thereby promoting Akt activation [33,34]. The activated Akt can downregulate NADPH oxidase, a major source of ROS under both physiological and pathological conditions, via blocking mTOR phosphorylation thereby decreasing ROS generation [35]. This could be another likely mechanism whereby baicalein reduces ROS generation, other than the possibilities of scavenging ROS or inhibiting Fe^2+^-regulated radical production. It may also improve survival by modulating its downstream signaling, supported by previous reports that Akt activation can downregulate the proapoptotic molecule BAD and upregulate antiapoptotic molecules such as NF-κB, leading to inhibition of caspase 9 [36]; and can phosphorylate FoxO family members and increase eNOS activation, which are directly correlated with survival [11,37]. In addition to its effect on regulating Akt, baicalein has been demonstrated to activate PKC and Erk [38,39] and inhibit caspase-8, and so forth [40].

Besides the protective effect of baicalein on regulating survival signaling, the present study, for the first time, demonstrated that baicalein improves contractility recovery in stunned cardiomyocytes. Myocardial stunning is a hallmark of cardiac arrest, and stunned myocardium not only decreases brain perfusion, but also causes the collapse of cardiac function even hours after cardiac arrest, elevating morbidity and mortality [41]. The enhanced contractility recovery is a result of Akt-promoted PLB phosphorylation (Figure 5), a downstream target of Akt that regulates contractility [23]. This action is supported by a previous study demonstrating that PLB phosphorylation increases the activity of the L-type Ca^2+^ channel complex, a known mechanism through which inotropism may be enhanced [23]. These protections were diminished by additional API-2 administration, indicating that Akt mediates baicalein-inducedcontractility recovery.

Previous reports suggest that both baicalein and cooling activate Akt [11,17]. This provides an explanation as to why the combined treatments of baicalein and cooling had no synergistic effects, as they act on the same signaling pathway. Akt activation appears to be a common protecting signaling pathway in the heart, shared by intralipid emulsion [22], an agent used as a myocardial energy substrate after ischemic arrest and to treat drug-induced cardiotoxicity [42,43,44]. Baicalein is superior to intralipid emulsion, because the delay of baicalein treatment starting at 20 min of reperfusion still preserves Akt activation and protection, while intralipid emulsion loses its cardiac protection when delayed. This beneficial effect may also be, in part, attributed to baicalein scavenging properties, which would make baicalein more attractive in SCA resuscitation and an ideal candidate as an adjuvant of cooling treatment in post-I/R resuscitation, which provides remarkable neuroprotection in clinical applications [45,46].

This study has its limitations. We used cardiomyocyte stunning and I/R models that simulate reversible stunned myocardial injury and irreversible tissue damage such as cell death. However, both of them do not model the physiological system of clinical cardiac arrest. In addition, the improvement of neurological function following cardiac arrest is critical to cardiac arrest outcome. This study demonstrated evidence that baicalein benefits cardiac function recovery, but its effects on brain function in the setting of delayed cooling are unclear. Thus, further studies in animal models are needed to assess the therapeutic potential of baicalein in cardiac arrest resuscitation.

## 4. Materials and Methods

### 4.1. Ethics Statement

The investigation conforms to the Guide for the Care and Use of Laboratory Animals, published by the National Institute of Health (NIH Publication No. 85-23, revised 1996). The procedure for cardiomyocyte isolation was approved by the University of Illinois at Chicago’s Institutional Animal Care and Use Committee (Permit Number: 11-198 (15 November 2011) and 14-165 (21 October 2016)).

### 4.2. Mouse Cardiomyocyte Culture

Primary mouse ventricular cardiomyocytes were prepared from hearts of 1- to 2-day-old C57BL6 mice (Jackson Laboratories, Bar Harbor, ME, USA) as previously described [11]. Experiments were performed on the culture day 5–7. 2-deoxy-glucose, trypsin inhibitor, bovine serum albumin, laminin, penicillin, and vitamin B_12_ were purchased from Sigma Chemical Co. (St. Louis, MO, USA).

### 4.3. Cooling Protocol in I/R and Stunning Models

A mouse cardiomyocyte I/R model and stunning model were used to study the cell viability and contractile alteration, respectively. In the I/R model, cardiomyocytes were equilibrated for 30 min and subjected to 90 min simulated ischemia followed by 180 min reperfusion. In the stunning model, cardiomyocytes were equilibrated for 30 min and subjected to 30 min simulated ischemia followed by 90 min reperfusion. Experiment groups included: (1) I/R, 90 min simulated ischemia followed by 180 min reperfusion. Cells were equilibrated 30 min (Eq prior to the ischemic insult; (2) I/R + Intraischemic cooling (I/R + IC). Cooling (32 °C) was initialed at the last 20 min ischemia and continued into 60 min reperfusion for a total of 80 min. Cells were then rewarmed to 37 °C; (3) I/R + Delayed cooling R5 (DC-R5). Cooling was delayed and initiated at 5 min reperfusion for a total 80 min; (4) I/R + Delayed cooling R10 (DC-R10). Cooling was delayed and initiated at 10 min reperfusion for a total 80 min; (5) I/R + Delayed cooling R15 (DC-R15). Cooling was delayed and initiated at 15 min reperfusion for a total 80 min; (6) I/R + Delayed cooling R20 (I/R + DC-R20). Cooling was delayed and initiated at 20 min reperfusion for a total 80 min; (7) I/R + DC-R20/BC. Cooling was delayed and initiated at 20 min reperfusion for a total 80 min. Baicalein (BC, 25 µM) was administrated at the start of reperfusion; (8) DC-R20/BC-C. Cooling was delayed and initiated at 20 min reperfusion for a total 80 min. Baicalein was administrated at the start of cooling; (9) I/R + BC alone. Baicalein was administrated at reperfusion; (10) I/R + IC/BC. Cooling was initiated at the last 20 min ischemia and continued into 60 min reperfusion for a total of 80 min, as in group 2. Baicalein was administrated at the start of reperfusion; (11) DC-R20/BC + API-2. Cells were preincubated with API-2 (10 µM) for 1 h prior to ischemic insult. Some of the protocols were described in Figure 1A. In the stunning model, cells were equilibrated for 30 min and then subjected to 30 min simulated ischemia, and 90 min reperfusion followed by 10 min isoproterenol (10 µM) to evaluate the reserve capacity of contractility activated by β-adrenergic stimulation. The protocols (Figure 1B) include: (1) Stunning. Cardiomyocytes were equilibrated for 30 min and subjected to 30 min simulated ischemia followed by 90 min reperfusion; (2) Stunning + DC-R20. Cooling was initiated at 20 min reperfusion; (3) Stunning + DC/BC. Baicalein was administrated at the reperfusion for 90 min.

### 4.4. Cell Viability

Cell death was assessed using propidium iodide (PI, 5 µM) as reported previously [10,11]. Briefly, fluorescence images were obtained (excitation 555 nm/emission 605 nm). Cells were permeabilized at R180 with digitonin (300 µM) for 45 min. Cell death was expressed as the PI fluorescence relative to the maximal value seen after digitonin exposure at the end of reperfusion (100%) [47]. All reported time values are as minutes following their specific treatment. Cell death was measured in 3 separate regions per cover slip. Propidium iodide was obtained from Sigma Chemical Co. (St. Louis, MO, USA). Baicalein was obtained from Sigma Chemical Co. (St. Louis, MO, USA). API-2 was purchased from EMD Chemicals (Philadelphia, PA, USA).

### 4.5. Hypoxia Box

Hypoxia chamber was used to simulate ischemic condition as previously reported [15] for collecting samples for molecular analysis. Briefly, the cells were equilibrated for 30 min in standard balanced salt solution (BSS), then placed into a hypoxic chamber (37 °C, 1% O_2_, 20% CO_2_ and 79% N_2_) with balanced salt solution immediately changed to the bubbled ischemia BSS for 30 min in the hypoxic chamber. The cells were subsequently removed from hypoxia chamber with an immediate change of the ischemic BSS to standard BSS and incubated at 37 °C, 21% O_2_, and 5% CO_2_ for 30 min. Then cells were harvested for molecular analysis. The ischemic conditions were simulated (PO_2_ 3–5 Torr, PCO_2_ 144 Torr and pH 6.8) and verified as previously reported [47].

### 4.6. Measurement of Intracellular ROS

The 6-carboxy-2′,7′-dichloro-dihydrofluorescein diacetate (6-carboxy-H_2_DCFHDA, 1 µM) was used to measure intracellular ROS at 90 min of reperfusion (R90) as previously described [11]. This nonfluorescent cell permeable dye is oxidized to a highly fluorescent carboxy-dichlorofluorescein (DCF). The DCF fluorescence was measured at excitation 488 nm/emission 520 nm and expressed as arbitrary units (a.u.) [11]. 6-carboxy-2′,7′-dichlorodihydrofluorescein diacetate (6-carboxy-H_2_DCFDA) was obtained from Sigma Chemical Co. (St. Louis, MO, USA).

### 4.7. Western Blot Analysis

Samples were collected at 30 min of reperfusion (R30) and processed as previously described [11]. In brief, cells were lysed in lysis buffer. Samples were resolved on a 10% SDS-Page gel, transferred to a nitrocellulose membrane and incubated with primary antibodies against p-Akt (T308 and S473), p-JNK, p-phospholamban, or α-tubulin. The membranes were further incubated with HRP antibodies, and visualized by SuperSignal. Unless otherwise noted, lysates were analyzed at R30.

### 4.8. Measurement of the Contractile Velocity

Movement of synchronously contracting cardiomyocytes was recorded with phase-contrast microscopy following the same field of cells over time using a Nikon ECLIPSE T*i* inverted phase/fluorescent microscope (Nikon Instruments Inc., Melville, NY, USA) as previously described [22]. Speckle Image Velocimetry [48] was used to quantify contraction velocity with custom written MATLAB software (Mathworks, Natick, MA, USA). Isoproterenol was obtained from Sigma Chemical Co. (St. Louis, MO, USA).

### 4.9. Statistical Analysis

Results are expressed as mean ± SE. A field of ~500 cells was observed in each experiment. To ensure reproducibility, each set of experiments consisted of replicates (*n*) generated from multiple batches of cells. For comparison among the different treatment groups, a Student *t*-test and one-way ANOVA were used with posthoc examination by Tukey’s test. For serial measurement data, a two-way repeated measures ANOVA was applied with Tukey’s posthoc analysis. A *p* value of < 0.05 was considered statistically significant.

## 5. Conclusions

In conclusion, the present study, using two cardiomyocyte models of I/R and stunning, illustrated that baicalein exerts cardioprotection in the context of delayed cooling by not only decreasing cell death and ROS production, but also improving cell contractility. Further, baicalein was demonstrated to increase phospholamban phosphorylation through Akt activation, thereby improving contractility recovery. These results suggest that baicalein may be used as an adjuvant of cooling for reducing I/R-induced injury.
